# Estimating below‐canopy light regimes using airborne laser scanning: An application to plant community analysis

**DOI:** 10.1002/ece3.5462

**Published:** 2019-07-26

**Authors:** Florian Zellweger, Andri Baltensweiler, Patrick Schleppi, Markus Huber, Meinrad Küchler, Christian Ginzler, Tobias Jonas

**Affiliations:** ^1^ Swiss Federal Institute for Forest, Snow and Landscape Research WSL Birmensdorf Switzerland; ^2^ Forest Ecology and Conservation Group, Department of Plant Sciences University of Cambridge Cambridge UK; ^3^ WSL Institute for Snow and Avalanche Research SLF Davos Dorf Switzerland

**Keywords:** airborne light detection and ranging LiDAR, beta diversity, biodiversity, canopy structure, Ellenberg indicator value, forest biodiversity, hemispherical photography, light availability, microclimate, remote sensing

## Abstract

Light is a key driver of forest biodiversity and functioning. Light regimes beneath tree canopies are mainly driven by the solar angle, topography, and vegetation structure, whose three‐dimensional complexity creates heterogeneous light conditions that are challenging to quantify, especially across large areas. Remotely sensed canopy structure data from airborne laser scanning (ALS) provide outstanding opportunities for advancement in this respect. We used ALS point clouds and a digital terrain model to produce hemispherical photographs from which we derived indices of nondirectional diffuse skylight and direct sunlight reaching the understory. We validated our approach by comparing the performance of these indices, as well as canopy closure (CCl) and canopy cover (CCo), for explaining the light conditions experienced by forest plant communities, as indicated by the Landolt indicator values for light (*L*_light_) from 43 vegetation surveys along an elevational gradient. We applied variation partitioning to analyze how the independent and joint statistical effects of light, macroclimate, and soil on the spatial variation in plant species composition (i.e., turnover, Simpson dissimilarity, *β*_SIM_) depend on light approximation methodology. Diffuse light explained *L*_light_ best, followed by direct light, CCl and CCo (*R^2^* = .31, .23, .22, and .22, respectively). The combination of diffuse and direct light improved the model performance for *β*_SIM_ compared with CCl and CCo (*R^2^* = .30, .27 and .24, respectively). The independent effect of macroclimate on *β*_SIM_ dropped from an *R* ^2^ of .15 to .10 when diffuse light and direct light were included. The ALS methods presented here outperform conventional approximations of below‐canopy light conditions, which can now efficiently be quantified along entire horizontal and vertical forest gradients, even in topographically complex environments such as mountains. The effect of macroclimate on forest plant communities is prone to be overestimated if local light regimes and associated microclimates are not accurately accounted for.

## 1 INTRODUCTION

Light—the visible range of the solar radiation spectrum—is one of the most important limiting factors in forests driving various ecological processes, such as plant establishment, growth, and survival (Kimmins, 2004). In forests, the quantity and quality of light and its spatial and temporal distribution is largely driven by canopy structure and composition, as well as topographic position (Canham, Finzi, Pacala, & Burbank, 1994; Lieffers, Messier, Stadt, Gendron, & Comeau, 1999). Canopy gaps, for instance, allow direct sunlight and associated energy to penetrate the canopy, resulting in sunflecks that move along the forest floor as the day progresses. In combination with nondirectional diffuse skylight, these sunflecks produce a small‐scale light regime that is highly variable in space and time, causing heterogeneity in microenvironmental conditions and resource availability that are vital to the dynamics and coexistence of forest species (Bazzaz & Wayne, 1994). Thus, estimating below‐canopy light conditions and analyzing their effects on biodiversity are crucial tasks for ecologists. By providing highly detailed and area‐wide available 3D forest structure data, airborne laser scanning (ALS) has opened new opportunities for advancement in this field.

Below‐canopy light conditions and the associated amount of energy available to forest organisms are commonly approximated by visually estimating canopy structure attributes, such as canopy cover and canopy closure (Jennings, Brown, & Sheil, 1999; Lieffers et al., 1999). Canopy cover is frequently referred to as the proportion of the forest floor covered by the vertical projection of the tree crowns, whereas canopy closure—sometimes also referred to 1 minus sky view fraction—refers to the proportion of the sky hemisphere obscured by vegetation when viewed from a single point (Jennings et al., 1999; Korhonen & Morsdorf, 2014). Due to the angular viewpoint, canopy closure is expected to represent the light conditions more accurately than canopy cover. Indeed, Alexander, Moeslund, Bøcher, Arge, and Svenning (2013) used averaged Ellenberg indicator values for light to show that canopy closure is an ecologically more meaningful proxy for understory light conditions than canopy cover. Moreover, canopy cover and canopy closure can be used to estimate functional variables such as Leaf Area Index (LAI), which is often applied to describe light absorption by trees (Binkley, Campoe, Gspaltl, & Forrester, 2013; Schleppi & Paquette, 2017).

Field observations of canopy structure are tedious and restricted in spatial coverage, making them inefficient for area‐wide, high‐fidelity mapping of light regimes along entire horizontal and vertical forest profiles. Therefore, detailed spatial data about light regimes are often missing in forest sciences, for example, forest biodiversity assessments using ecological niche models (Peterson et al., 2011), despite the importance of small‐scale light regimes and associated microclimatic conditions for organisms dwelling beneath canopies. Attempts to fill this gap are spurred by the rapidly increasing availability of highly detailed three‐dimensional forest structure data from remote sensing, airborne laser scanning (ALS) in particular (Davies & Asner, 2014; Lefsky, Cohen, Parker, & Harding, 2002; Zellweger, Frenne, Lenoir, Rocchini, & Coomes, 2019).

Airborne laser scanning approaches to extract canopy structure metrics to approximate light conditions and LAI have been thoroughly studied (Alexander et al., 2013; Korhonen, Korpela, Heiskanen, & Maltamo, 2011; Majasalmi, Korhonen, Korpela, & Vauhkonen, 2017; Moeser, Roubinek, Schleppi, Morsdorf, & Jonas, 2014; Morsdorf, Koetz, Meier, Itten, & Allgoewer, 2006; Parker, Lefsky, & Harding, 2001; Solberg et al., 2009). While area‐based measurements, such as canopy cover, are frequently used (Morsdorf et al., 2006; Zellweger et al., 2015), point‐based measurements, such as canopy closure, are computationally more demanding and less often used, despite them being ecologically more relevant (Alexander et al., 2013). Similarly, voxel‐based ray tracing techniques have been successfully applied to estimate below‐canopy light conditions and associated microclimate conditions (Musselman, Pomeroy, & Link, 2015; Tymen et al., 2017).

Although the detailed representation of the canopy geometry in ALS point clouds enable the quantification of both diffuse light and direct light incidence, that is, by tracking the sun on a daily and seasonal basis (Chazdon & Field, 1987; Schleppi & Paquette, 2017), currently used ALS light approximation methods do not differentiate between diffuse and direct light. This is a shortcoming because these two components show different spatial and temporal distribution patterns and vary in terms of light quantity and associated energy input (Lieffers et al., 1999), providing complementary information for analyzing ecological processes beneath tree canopies. Diffuse radiation, for example, is expected to be more closely related to net photosynthesis than direct solar radiation because of a more efficient distribution of nonsaturating light conditions for photosynthesis (Law et al., 2002). Increased direct solar radiation input, however, may be associated with higher local temperatures and increased respiration demands due to a higher vapor pressure deficit (Kimmins, 2004). Moreover, many light approximations methods employing ALS make none or crude assumptions about terrain shading effects. Local and regional terrain attributes, such as slope aspect and shading of nearby mountains, are key determinants of local radiation regimes, especially in mountain areas (Bramer et al., 2018). Integrative ALS‐based approaches that simultaneously account for both canopy and topography effects on local light regimes are rarely available but urgently required to further our understanding of how microclimates affect biodiversity dynamics and ecosystem responses to global change (Zellweger et al., 2019).

A promising solution to overcome these limitations is to approximate light regimes from synthetic hemispherical photographs derived from ALS point clouds and a regional terrain model (Bremer, Wichmann, & Rutzinger, 2017; Moeser et al., 2014; Varhola, Frazer, Teti, & Coops, 2012). We therefore extended a previously developed tool by Moeser et al. (2014) to generate such synthetic hemispherical images to include the following new properties: (a) topographic shading and accounting for the accurate position of canopy elements on sloping terrain and (b) distinguishing between diffuse and direct light reaching the forest understorey. We validated our approach by comparing the performance of diffuse and direct light, as well as canopy closure and canopy cover, for explaining the average Landolt indicator values for light (*L*_light_)—a regional adjustment of the Ellenberg indicator value for light (Landolt et al., 2010) —derived from plant surveys. Then, we compared the independent and joint statistical effect of our light approximations, macroclimate, and soil characteristics on the spatial variation of vascular plant species composition, that is, beta diversity, to shed light into the relative importance of microenvironmental factors for structuring forest plant communities.

## 2 DATA AND METHODS

### 2.1 ALS data and methods

We used ALS data acquired during 2010 and 2015, mostly during leaf‐off conditions. The minimum ALS point return density—from now on referred to point density—for all our study plots (see below) was 10 or more points per square meter. The raw data were preprocessed using a suite of *LAStools* algorithms (Isenburg, 2016) to classify the ALS points into terrain and vegetation and to normalize the vegetation point heights to calculate canopy cover (see below).

#### 2.1.1 Tool for ALS‐derived hemispherical images

**Figure 1:**
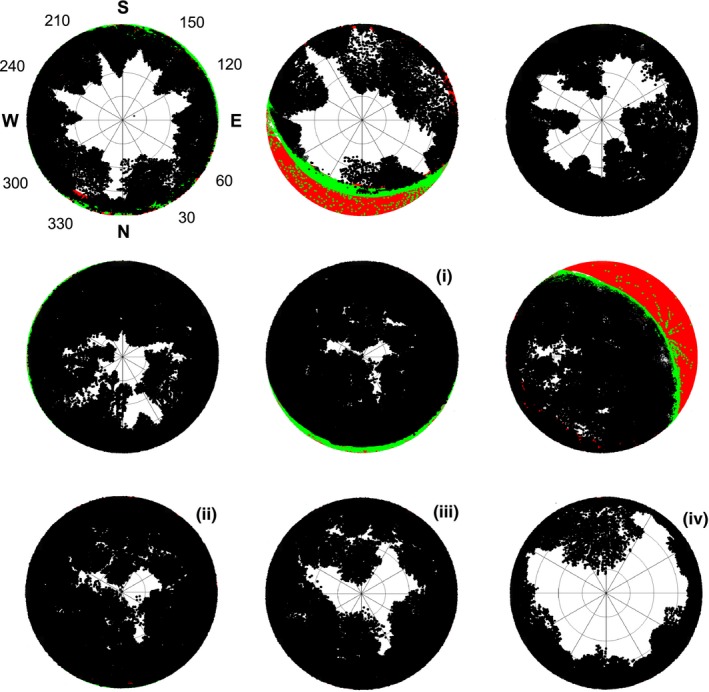
Synthetic hemispherical images derived from non‐normalized ALS point clouds and a digital elevation model. The orientation and angles (°) are all the same as shown for the top‐left image. The top and middle rows show images generated at 1 m height above ground for six stands along a canopy closure gradient. Macro‐ and microterrain shadings are shown in red and green, respectively. Images i to iv illustrate a vertical gradient of canopy structure in the same stand, and the viewpoints above ground of ii to iv are at 7.5, 15, and 25 m, respectively

To mimic hemispherical photographs from ALS data, we developed a tool that builds upon the methods described by Moeser et al. (2014) and extend this approach to account for topography shading, as well as to distinguish between nondirectional diffuse skylight and direct sunlight. To take a point‐based angular viewpoint, all raw data were transferred into a spherical coordinate system where the traditional Cartesian coordinates (*X*, *Y*, *Z*) were converted into a distance from the origin (*r*), the elevation angle (theta), and the azimuth angle (phi). Each return within the ALS point cloud data was then printed in a polar coordinate system according to *r* and phi, thereby mimicking a hemispherical image taken using a circular fisheye lens with an equiangular projection. To conceptually distinguish between near and far distance canopy elements, each return was printed as a black point the size of which linearly decreasing with *r* (Moeser et al., 2014); thus, black points can then overlap as leaves would overlap on a real hemispherical picture. We applied the settings suggested by Moeser et al. (2014), who included all points within a radius of 100 m. When compared to real hemispherical photographs, they achieved best results with a print size of 7 pixels for the nearest, and 0.5 pixels for the farthest canopy elements, respectively. To account for topography shading, we additionally calculated the horizon line from a 100‐m digital terrain model and converted it into respective theta values in terms of phi. On the synthetic hemispherical image, all elevations below this horizon line were subsequently masked out (Figure 1, red areas). To further account for local terrain, also ground returns were printed on the image if located above the camera position (Figure 1, green points). Note that since we used ALS returns in true coordinates (as opposed to terrain‐corrected canopy height data), trees on the uphill slope would plot more toward the image center compared with trees on the downhill slope, which resulted in a more realistic representation of the canopy, particularly in steep terrain.

#### 2.1.2 Image analysis

The obtained synthetic hemispherical pictures were analyzed with image analysis software, that is, *Hemisfer*, version 2.2, (Schleppi, Conedera, Sedivy, & Thimonier, 2007; http://www.schleppi.ch/hemisfer/). The canopy closure was calculated as the solid‐angle proportion of the hemispherical vault that is obscured by canopy, or topography in case of terrain shading. The light regime at the site was calculated for the growing season (May to September), using the standard parameters of Hemisfer for the light climate. These parameters are the atmospheric transmission, that is, 40% as direct radiation + 20% as diffuse radiation, with barometric correction according to the altitude. Further, the model of standard overcast sky (SOC) according to Steven and Unsworth (1980) was used with the parameter *b* equal to 1. Based on these parameters, the software generates an incoming radiation from the whole sky vault (i.e., diffuse radiation, herein referred to as diffuse light) that varies over time and elevation above the horizon. The hemispherical picture is then used as a mask to limit this radiation to those directions that are not blocked by the vegetation. The same mask is also used for the direct solar radiation (herein referred to as direct light) except that it applies at any time only to the disk representing the virtual position of the sun on the picture. Both the diffuse and direct radiation were reported as a proportion of the radiation that would fall at the same latitude and altitude but on a flat ground without vegetation, that is, without the mask representing the canopy. These proportions are called diffuse light index and direct (beam) light index. Along with the canopy closure, these indexes are the three metrics that we derived from the image analysis.

Visual inspection of the ALS‐derived images suggests a very realistic representation of canopy structure and previous validation carried out by Moeser et al. (2014) using real hemispherical photographs and radiometer measurements further supports this. Their study, conducted in the same type of forest stands as we analyze here, revealed an excellent agreement between canopy closure derived from ALS images and canopy closure derived from real photographs (*r* = .923, *n* = 112). Moreover, the sum of diffuse and direct shortwave radiation measured with 10 radiometers at three sites was highly correlated with the potential incoming solar radiation estimated from hemispherical ALS images, with correlation coefficients ranging from .84 to .92 (Moeser et al., 2014). Similar correlation coefficients between hemispherical photographs and in situ light measurements have also been reported in other studies (Chazdon & Field, 1987; Hardy et al., 2004).

### 2.2 Application for plant community analysis

#### 2.2.1 Study area and sample plots

This study was conducted in Switzerland, Central Europe. The majority of Swiss forests are managed, mainly for timber, but other forest ecosystem services such as biodiversity, recreation, and protection from gravitational natural hazards are also considered in the management plans. We used data from 43 circular, 200 m^2^, conifer‐dominated plots distributed on a 4 × 4 km grid of the Swiss National Forest Inventory (NFI). The coordinates of the plots were measured with a differential GNSS, rendering an average horizontal accuracy of 1 m. The plots were selected according to the availability of recent ALS data with a point density of at least 10 points per square meter. The elevation of the plots ranges from 378 to 1,774 m above sea level, with a median of 879 m above sea level. Topographic slope and aspect varied considerably among the plots, with slope values ranging from 2.4 to 51.8 degrees (median = 20.0) and aspect values covering all aspects. Within the boundaries of a temperate humid climate, the mean annual temperature and precipitation across our study plots range from 3.1 to 9.5°C (median = 7.1°C) and 846 to 1,748 mm (median = 1,235 mm), respectively.

#### 2.2.2 Vegetation data

Understory vegetation surveys on all 43 plots have been carried out during the years 2008 and 2011. The sampling was performed by professional botanists, who applied the Braun‐Blanquet scheme to estimate the abundance of all vascular plants and bryophytes growing on the soil (Braun‐Blanquet, 1964; Küchler, Küchler, Bedolla, & Wohlgemuth, 2014). The median time gap between the vegetation and ALS surveys was 4 years. Because natural dynamics occur relatively slowly in the coniferous and coniferous dominated stands that we studied and because none of the plots have been harvested since the beginning of the vegetation surveys, we expected that the time gap between the two surveys did not significantly affect our main results and conclusions (see Section 3).

From the species data, we calculated two dependant variables: (a) the abundance‐weighted average Landolt indicator value for light (*L*_light_) and (b) the Simpson dissimilarity (*β*_SIM_), expressing the turnover component the spatial variation in species composition, that is, beta diversity (Baselga & Orme, 2012). Landolt indicator values are an adapted version of Ellenberg indicator values for Switzerland that improve the representation of the regional‐specific conditions (Landolt et al., 2010). Weighted Landolt indicator values were calculated for the entire plant community, applying the abundances as weight. These indicator values indicate the light conditions experienced by the herb‐layer community and are thus expected to be suitable to validate whether the outputs of our tool represent ecologically meaningful information (Diekmann, 2003). *β*_SIM_ was calculated based on a pairwise dissimilarity matrix of the plot‐based plant community compositions, following the methods proposed by Baselga and Orme (2012). We focussed on species turnover and did not analyze the nestedness component of beta diversity because our aim was to analyze the change of community composition along environmental gradients (light, climate, soil) (Vellend, 2001).

#### 2.2.3 Below‐canopy light regime variables

We used four below‐canopy light proxies as explanatory variables, that is, diffuse and direct light, canopy closure, and canopy cover. The indices of diffuse and direct light, as well as canopy closure were calculated from the ALS‐derived hemispherical images taken at 1 m above ground, as described above. To represent the light conditions in each plot, we generated 16 images per plot, based on a grid with a mesh size of 4 m. Canopy cover was calculated based on the normalized (terrain‐corrected) ALS point cloud as determined by the *lasheight* tool (Isenburg, 2016) and defined as the proportion of all first returns classified as vegetation above 1 m relative to all first returns. We used the *lascanopy* tool (Isenburg, 2016) to calculate a 4 m mesh size‐raster of this variable. To define canopy cover, we then extracted all pixel values within each of our field plots where the central point of the pixel was within the plot perimeter. We also calculated canopy cover based on the point cloud clipped out with the circular plot polygons, but the analysis revealed the same results as for the 4 m raster approach; thus, we used the raster.

To test how well our light proxies work for representing below‐canopy light conditions experienced by the plant communities, we used the log‐transformed values of each light proxy to calculate a mean for each plot and analyzed their performance in explaining the average Landolt indicator values for light (*L*_light_, see below) derived from plant surveys. Log‐transformed light availability is expected to be linearly related to Landolt indicator values, which range from 1 to 5, increasing approximately 2‐ to 3‐fold in light availability at each step.

To test for effects on beta diversity (*β*_SIM_), we considered the diffuse and direct light indices into one group and treated canopy closure and canopy cover separately. In addition to the mean light conditions for each plot, we also considered the spatial heterogeneity of light conditions, as represented by the standard deviation of each light proxy, because both the mean and spatial heterogeneity of local light conditions are expected to drive variation in plant community composition (Bazzaz & Wayne, 1994; Zellweger, Roth, Bugmann, & Bollmann, 2017). Although combining diffuse and direct light into one group of variables increases the number of variables tested, it is ecologically sensible as these two light components determine the light regime below canopy, which as an entity is expected to be ecologically more relevant for structuring plant communities than commonly derived canopy closure and canopy cover metrics.

#### 2.2.4 Macroclimate variables

Climate was represented by the number of degree days above a threshold of 3°C and the precipitation sum (mm) during the growing season (April to September). Temperature and precipitation layers with a 100‐m resolution were interpolated using DAYMET (Thornton, Running, & White, 1997) based on mean daily measurements of all available recording stations outside forests (ca. 300, www.meteosuisse.ch) and a digital elevation model (www.swisstopo.ch). Our climate data thus represent free‐air conditions derived from standardized weather stations, neglecting microclimatic variation brought about by canopy structure. We averaged the temperature and precipitation data using all data of the years 1981 to 2010; see Zellweger et al. (2016) for details.

#### 2.2.5 Topography and soil pH variables

We used three variables to represent soil characteristics at each plot: topographic wetness index (TWI), topographic position index (TPI), and topsoil pH. TWI describes the lateral water flow based on the specific upslope draining area divided by the tangent of the locale slope (Sørensen & Seibert, 2007). TPI describes the exposure of a site in relation to the surrounding terrain, where positive index values represent ridges and hilltops and negative values sink (Zimmermann & Roberts, 2001). Both TWI and TPI are proxies for edaphic run‐off processes and are thus expected to be related to soil water content, texture, and nutrient availability. TWI was computed using the SAGA GIS hydrology module (Olaya & Conrad, 2009), and TPI was calculated following the methods described in Zimmermann and Roberts (2001). The spatial resolution of the used digital elevation model was 5 m, which has been shown to be more adequate to quantify topographic effects on soil characteristics relevant to plants compared with coarser resolutions (Camathias, Bergamini, Küchler, Stofer, & Baltensweiler, 2013; Pradervand, Dubuis, Pellissier, Guisan, & Randin, 2014). Topsoil pH was derived from the nationally available layer that was interpolated by combining topsoil pH samples collected within the Swiss National Forest Inventory and a digital map depicting topography and geological parent material; further details are described in Zellweger et al. (2015).

### 2.3 Statistical analysis

We used linear regression models to analyze the performance of each of our ALS‐based proxies for below‐canopy light conditions for explaining *L*_light_. Increased model performance would thus indicate a more realistic representation of the light conditions experienced by the plant community on the forest ground.

To assess the independent and shared effects of different light proxies, macroclimate, and topography/soil on *β*_SIM_, we applied distance‐based redundancy analysis (db‐RDA; Legendre & Anderson, 1999) and variation partitioning based on the adjusted *R* ^2^ (Borcard, Legendre, & Drapeau, 1992). In other words, we tested how much of the variance in *β*_SIM_ can be explained by each environmental variable group separately, as well as by the combinations of different variable groups. db‐RDA and variation partitioning analyses were performed using the *capscale* and *varpart* functions, respectively, all implemented in the R package *vegan* (Oksanen et al., 2015). We performed variation partitioning using three groups of below‐canopy light proxies separately, as described above (i.e., diffuse and direct light, canopy closure, and canopy cover). This allowed us to test and analyze whether (a) our tool and the derived light regimes improve *β*_SIM_ predictions compared with canopy closure and canopy cover and (b) whether the independent and joint effects of light, macroclimate, and topography/soil depend on the light sampling methodology. All analyses were done in the R statistical programming language (R Core Team, 2015).

## 3 RESULTS

### 3.1 ALS proxies for below‐canopy light conditions

**Figure 2:**
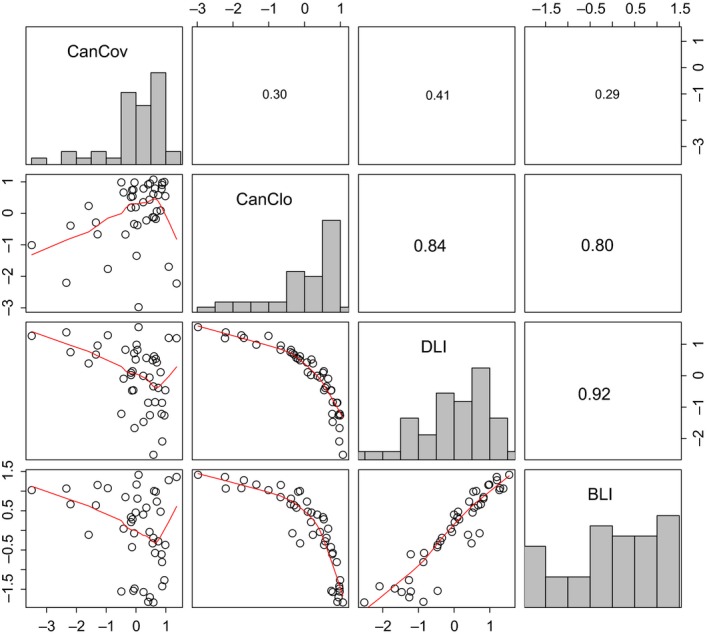
Correlation matrix and histograms of canopy cover (CanCov), canopy closure (CanClo), diffuse light index (DLI), and direct light index (BLI). The upper panel shows the absolute correlations (Pearson's correlation coefficient)

**Figure 3:**
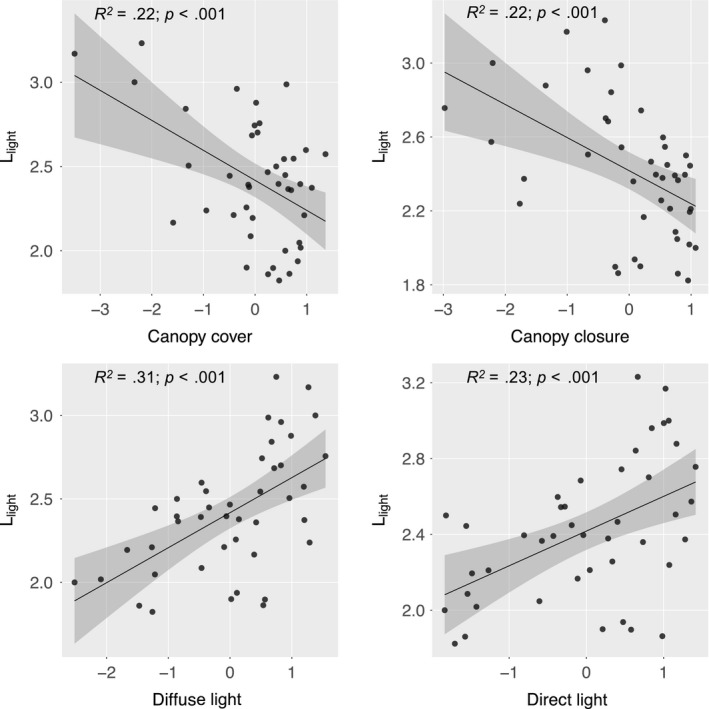
Linear regressions with confidence intervals of the relationships between log‐transformed below‐canopy light approximations and the mean Landolt indicator value for light (*L*_light_) derived from 43 vegetation surveys

Diffuse light was strongly correlated with direct light (Pearson *r* = .92) and canopy closure (*r* = .84), which showed a lower correlation with direct light (*r* = .80; Figure 2). Canopy cover was weakly correlated with the other three light metrics. Among the light proxies tested, diffuse light explained *L*_light_ best, with a *R* ^2^ value of .31 (Figure 2). The correlation between *L*_light_ and the light metrics was all significant, with a similar *R* ^2^ value for direct light, canopy closure, and canopy cover. The sum of diffuse and direct light, that is, the global radiation, showed a weaker relationship to *L*_light_ than diffuse light, despite the strong correlation between diffuse light and global radiation (Figures S1 and S2). To test whether the time gap between the ALS and vegetation surveys affected the above results, we checked whether the number of years between the surveys was correlated with the residuals from the regression models presented in Figure 3. None of the correlations were significant (*p* > .05), suggesting that the time gap between the surveys did not significantly affect our results and conclusions.

### 3.2 Effects on plant species turnover (*β*_SIM_)

**Figure 4:**
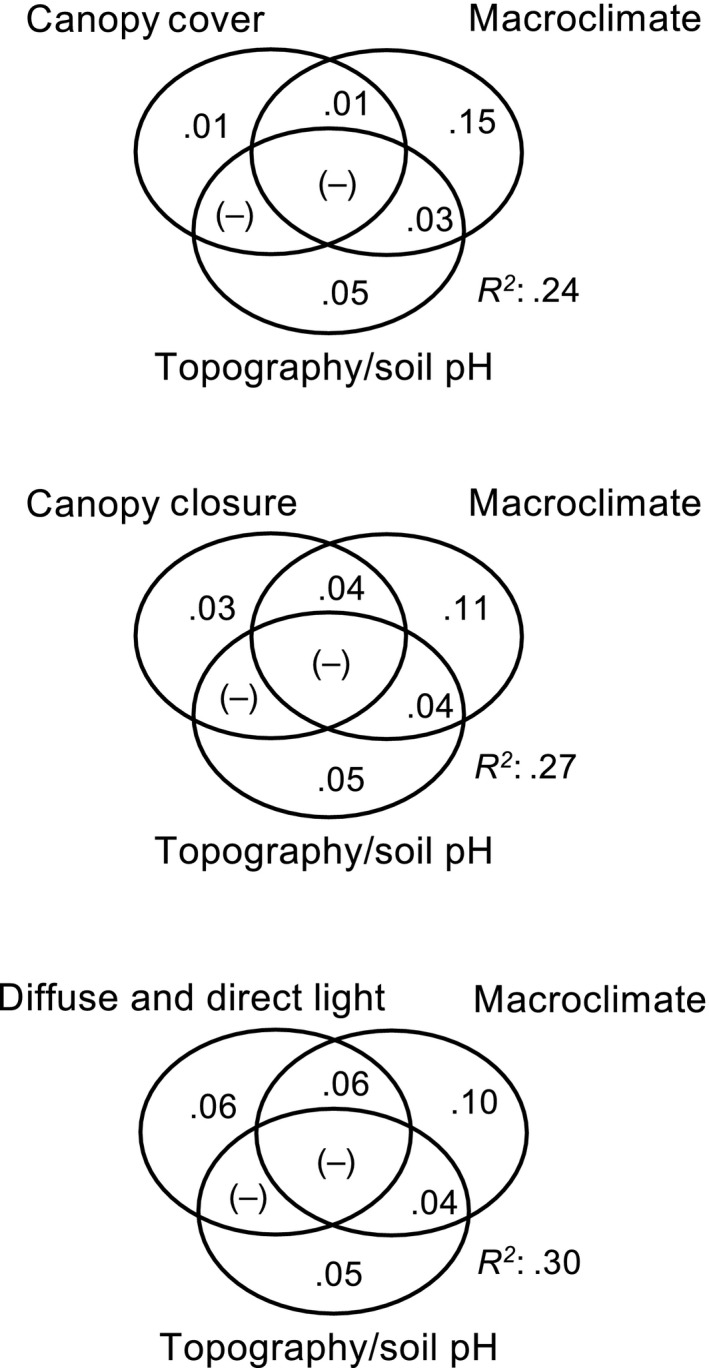
Results from variation partitioning based on distance‐based redundancy analysis (db‐RDA) relating the proxies for below‐canopy light conditions, macroclimate (expressed as degree days and precipitation derived from standardized, free‐air weather station data), and topography/soil pH (topographic position, topographic wetness, topsoil pH) to plant species turnover (Simpson dissimilarity, *β*_SIM_). The circles and their intersections show the independent and shared proportions explained variation (adjusted *R^2^*); negative values (−) are interpreted as zeros; and they represent cases where the explanatory variables explain less variation than random normal variables would (Legendre, 2008)

Variation partitioning between our light proxies, macroclimate, and soil characteristics revealed that including diffuse and direct light indices (i.e., the light regime) improved the overall model performance for explaining *β*_SIM_ compared with canopy closure and canopy cover, as indicated by *R* ^2^ values of .30, .27, and .24, respectively (Figure 4). The independent share of explained variation by diffuse and direct light was .06 and thus considerably higher than that of canopy closure (.03) and canopy cover (.01). Using the sum of diffuse and direct light, that is, the global radiation, resulted in lower *R* ^2^ values compared with the model that includes the effects of both diffuse and direct light (Figure S3). The shared portion of explained variation between the light proxies and macroclimate increased from .01 to .06 when diffuse and direct light indices were included instead of canopy cover. Likewise, the independent share of explained variation by macroclimate decreased from .15 to .10 when the light regime was included instead of canopy cover. There were no joint shares of explained variation between topography/soil pH and light proxies, and the independent and joint shares of topography/soil pH stayed constant across all three models (Figure 4).

## 4 DISCUSSION

We show that improved methods to calculate diffuse and direct light components from ALS‐based synthetic hemispherical images outperform conventional ALS‐based proxies for below‐canopy light conditions. The light conditions experienced by forest plant communities, as expressed by abundance‐weighted indicator values for light (Diekmann, 2003), were best explained by nondirectional diffuse skylight. This finding corroborates the expectation that net photosynthesis should be more strongly related to diffuse than to direct radiation, due to a more efficient distribution of nonsaturating light conditions for photosynthesis, lower vapor pressure deficit limitation to photosynthesis, and lower respiration associated with reduced temperature (Law et al., 2002). This finding is also in line with previous results from Alexander et al. (2013), who showed that point‐based ALS‐based approaches that take an angular viewpoint perform better in explaining understory light conditions than area‐based approaches, such as canopy cover. Together with the increasing availability and accessibility of spatial ALS data, integrative approaches that simultaneously account for both canopy shading and topographic effects on local radiation regimes will greatly facilitate and improve high‐fidelity mapping of light regimes and associated ecological niches in three‐dimensionally complex habitats such as mountains and forests.

Our methods have major advantages over field‐based methods that are tedious and much restricted in terms of spatial coverage, particularly along vertical canopy gradients that are not easily accessible. The flexibility of our approach may thus benefit canopy biodiversity research, as detailed data on light and available energy to canopy‐dwelling organisms are often missing. In general, incorporating detailed local light regimes into biodiversity research is promising because the scale at which environmental heterogeneity is quantified can be matched with the scale at which environmental heterogeneity is perceived by the focal species and individuals. This resolves a prominent shortcoming of frequently used, more coarsely resolved environmental datasets. For example, our approach may significantly improve the analysis of species' niches in forests by means of ecological niche models, where light availability and its spatial heterogeneity are currently underrepresented predictor variables (Peterson et al., 2011). Due to the interaction between incoming solar radiation and several microclimatic parameters (e.g., surface and air temperatures, air humidity, or soil moisture), it will be interesting to test the presented method for predicting microclimate by combining it with the growing number of microclimate sensor networks (Zellweger et al., 2019).

The application of our tool to study environmental effects on forest plant species beta diversity (turnover) revealed that the combination of diffuse and direct light, that is, the light regime derived from ALS‐based hemispherical images, considerably improved the model fit compared with canopy closure and canopy cover. Although both diffuse and direct light affect plant ecological niches via the availability of photosynthetically active radiation (PAR), plant niches may also be indirectly affected by direct light incidence via its effect on topsoil and air temperature, as well as humidity levels, which have been shown to be strongly related to total radiation across the forest gap‐understory continuum (Bazzaz & Wayne, 1994). As mentioned above, both diffuse and direct light are related to local environmental (microclimate) filters that significantly affect species assemblages, yet commonly used ALS‐based proxies for below‐canopy light conditions often neglect such nuances. Future attempts to approximate below‐canopy light conditions from ALS data should thus take full advantage of the canopy geometry provided by ALS and aim at thorough measurements of spatiotemporal light regimes.

We further found that the effect of macroclimate on plant beta diversity decreased when the diffuse and direct light indices were included instead of canopy closure or canopy cover. This is due to the fact that forest structure and related below‐canopy light availability are slightly positively correlated with altitude and thus negatively with the degree days sum. Correlations between forest structure and altitude are common in mountain regions, and in our dataset, they cause the joint effect of light and macroclimate on species turnover, but this aspect only emerges once the light regime is accurately accounted for. Thus, the importance of the macroclimate for driving dynamics in forest plant communities is prone to being overestimated if local light regimes are not accurately represented in the analysis. Despite this regional context, our results provide ample evidence that a more sophisticated representation of local light regimes improves insights into the different dimensions of environmental filtering of plant communities. This finding is in coincidence with the growing evidence for the global importance of microclimate in driving forest plant dynamics (De Frenne et al., 2013). It is furthermore interesting to note that the relative and joint effects of topography and soil characteristics stayed constant across all models with different light approximations, implying soil characteristic filter plant communities in a way that is truly independent of the light conditions. Taken together, this exemplifies that improved methods for quantifying local light regimes will further our understanding of the relative and joint effects of large‐ and local‐scale drivers of biodiversity, such as climate change and forest management effects on below‐canopy light conditions.

It is important to note that our approach is subject to a number of limitations. The results presented here are based on relatively high ALS point densities, that is, more than 10 points (return signals) per square meter. Using lower density ALS point clouds is certainly possible but would likely require a recalibration of the image output using real hemispherical photographs and an adjusted scaling factor for the print size of the ALS points as a function of distance (see Section 2.1.1). Similar limitations will also apply for using ALS data and derived synthetic hemispherical images that are strongly affected by the season during which they were collected, that is, during leaf‐off or leaf‐on season. This especially applies to deciduous forests, where ALS‐derived canopy characteristics can considerably vary depending on the time of acquisition of the ALS data (Wasser, Day, Chasmer, & Taylor, 2013). In evergreen forest, such as the ones studies here, biases due to seasonal changes in leaf area are expected to be lower (Wasser et al., 2013). Although the time gap between the ALS surveys and the vegetation surveys did not affect our main results and conclusion, it is important to consider that such time gaps can introduce a substantial analytical bias, especially in forest types and stages where changes in canopy structure occur at a faster rate.

A promising alternative to generate realistic hemispherical images from laser scans is to use terrestrial laser scanning (TLS; Bremer et al., 2017), but TLS provides point‐based samples and is thus not available continuously over larger areas. Moeser et al. (2014) further found that ALS‐derived hemispherical images tend to overestimate canopy closure under very dense canopies, probably because small canopy gaps are underrepresented in such conditions. Our approach also neglects potential effects of canopy composition on the light regime below the canopy. It has been found that light indices determined by hemispherical photographs may underestimate total PAR in stands dominated by very shade‐tolerant tree species because below such trees a considerable portion of PAR comes from beam enrichment, that is, beam radiation that is successively reflected downward by foliage (Canham et al., 1994). However, synthetically derived hemispherical images from ALS data have a number of advantages over traditional hemispherical photography. In addition to the great flexibility described above, these include no undesired illumination effects on the lens, no biases from camera settings, and no threshold needs to be chosen to differentiate sky from vegetation. Our approach is thus objective, reproducible, and much less time consuming than traditional hemispherical photography.

## CONFLICT OF INTEREST

None declared.

## AUTHORS' CONTRIBUTIONS

F.Z. conceived the ideas and designed the study in collaboration with T.J. and M.K. T.J., P.S., and C.G. wrote code for data processing and analysis. M.K. and A.B. provided data and valuable methodological comments. F.Z. conducted the analyses and wrote the manuscript, which was approved by all authors.

## Data Availability

The MATLAB code to derive hemispherical images from ALS data is on GitHub (https://github.com/Tobias-Jonas-SLF/Lidar2Hemi). Hemisfer is available online (http://www.schleppi.ch/hemisfer/), and there are a number of other open access tools available for hemispherical image analysis, some of them allowing for batch processing (Hall, Cote, Mailly, & Fournier, 2017).
