# Supplementary material for: Estimating below‐canopy light regimes using airborne laser scanning: An application to plant community analysis
Source: Ecol Evol. 2019 Jul 26;9(16):9149–59. doi: 10.1002/ece3.5462 (PMC6706208; doi:10.1002/ece3.5462)
Supplement: Supplementary file 1 [file ECE3-9-9149-s001.docx]

**Estimating below-canopy light regimes using airborne laser scanning: an application to plant community analysis**

Florian Zellweger, Andri Baltensweiler, Patrick Schleppi, Markus Huber, Meinrad Küchler, Christian Ginzler, Tobias Jonas

*Submitted to Ecology and Evolution*

**Supporting Information**

**Figure S1**. Correlation matrix and histograms of below-canopy light indices. CanCov: canopy cover; CanClo: canopy closure; DLI: non-directional diffuse light; BLI: direct sunlight; GLI: global radiation, i.e. the sum of DLI and BLI.

**
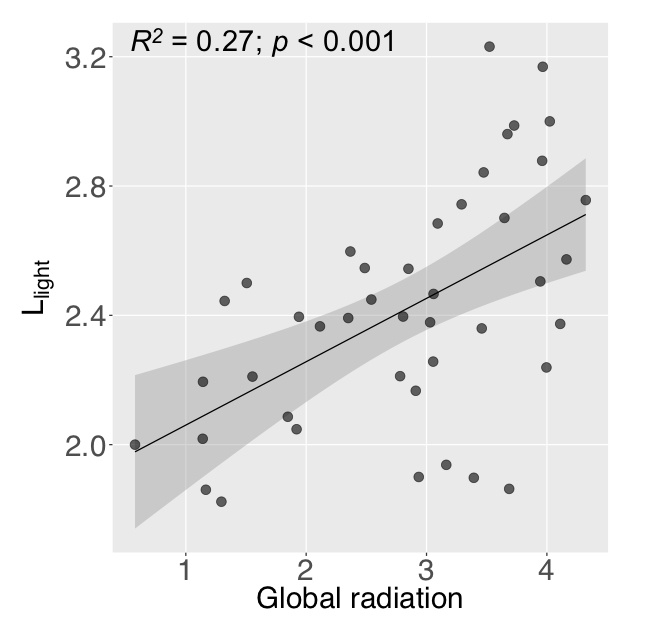
**

**Figure S2**. Linear regression with confidence intervals of the relationships between log-transformed global radiation index (i.e., the sum of non-directional diffuse sky light and direct sunlight) and the mean Landolt indicator value for light (L_light_) derived from 43 vegetation surveys.

**Figure S3**. Results from variation partitioning based on distance-based redundancy analysis (db-RDA) relating the global radiation index (i.e., the sum of non-directional diffuse sky light and direct sunlight), macroclimate (expressed as degree-days and precipitation derived from standardised, free-air weather station data) and topography/soil pH (topographic position, topographic wetness, topsoil pH) to plant species turnover (Simpson dissimilarity, β_SIM_). The circles and their intersections show the independent and shared proportions explained variation (adjusted *R^2^*); negative values (-) are interpreted as zeros; they represent cases where the explanatory variables explain less variation than random normal variables would (Legendre, 2008).
